# Novel applications of SIRT1 in Chronic Kidney Disease

**DOI:** 10.3389/fmed.2026.1922310

**Published:** 2026-09-23

**Authors:** Yucheng Han, Qianwei Wang, Xuanbing Pan, Lijuan Dai

**Affiliations:** 1 Graduate School, Heilongjiang University of Chinese Medicine, Harbin, China; 2 Department of Nephrology, First Affiliated Hospital of Heilongjiang University of Chinese Medicine, Harbin, China

**Keywords:** Chronic Kidney Disease, SIRT1, kidney fibrosis, inflammation, oxidative stress, autophagy, therapeutic target

## Abstract

Chronic kidney disease (CKD) has become a global public health issue, with progressive kidney fibrosis serving as a key pathological process leading to kidney failure, and current therapeutic approaches remain largely ineffective in reversing this progression. SIRT1, as an NAD⁺-dependent deacetylase, plays a central protective role in counteracting kidney fibrosis, inflammation, oxidative stress, and autophagic dysfunction by regulating multiple signaling pathways, and has emerged as a promising target in CKD treatment. This review systematically elaborates on the mechanisms regulating SIRT1 expression and activity in CKD, its protective roles in core pathological processes, and summarizes recent advances in targeting SIRT1 for CKD therapy. Functionally, SIRT1 antagonizes kidney fibrosis by deacetylating Smad3/Smad4 and inhibiting epithelial-mesenchymal transition; curbs inflammatory cascades through dual blockade of NF-κB transcriptional activity and NLRP3 inflammasome assembly; constructs a multi-layered antioxidant defense network by activating the Nrf2/ARE pathway, regulating FOXO transcription factors, and enhancing mitochondrial biogenesis; and restores autophagic flux to clear damaged organelles. Regarding complications, SIRT1 inhibits vascular calcification and improves kidney anemia by stabilizing HIF-1α to promote EPO synthesis and inhibiting ferroptosis. In terms of therapeutic strategies, the synthetic SIRT1 agonist SRT2104 has completed Phase I-II clinical trials with favorable safety profiles, but its pharmacokinetic limitations and the disconnect from preclinical effects pose barriers to clinical translation; NAD⁺ precursor strategies offer theoretical advantages but lack clinical evidence in CKD populations. In summary, SIRT1 integrates metabolic sensing, transcriptional regulation, and post-translational modification functions, serving as a key molecular hub linking upstream pathological signals to downstream organ damage in CKD. Precise modulation of SIRT1 activity may offer a novel multi-target synergistic therapeutic strategy for CKD.

## 1 Introduction

Chronic kidney disease (CKD) has become one of the major global public health issues, serving as both a direct cause of global morbidity and mortality and a major risk factor for cardiovascular disease (1). Diabetes and hypertension are the two leading causes of CKD. Studies have confirmed a very close association between CKD and the risks of death, cardiovascular events, and hospitalization (2). The pathophysiological process of CKD is a core progressive fibrosis process that begins with initial injury, passes through the critical link of hyperfiltration in surviving nephrons, triggering responses such as inflammation and RAAS activation, and ultimately leads to a large number of nephrons being replaced by scar tissue, loss of function, and the formation of a vicious cycle. Kidney fibrosis, particularly renal interstitial fibrosis, is the common final outcome of most progressive chronic kidney diseases. Renal fibrosis is also a reliable predictor of prognosis and a major determinant of kidney dysfunction.

Sirtuins are a group of evolutionarily conserved nicotinamide adenine dinucleotide (NAD⁺)-dependent class III histone deacetylases. The sirtuin family consists of seven members (SIRT1-7), among which sirtuin 1 (SIRT1) is the most extensively studied member (3–5). The expression and activity of SIRT1 are ubiquitously suppressed in CKD, and its loss of function is a key factor leading to various pathological processes of CKD (6). SIRT1 plays an important role in inflammation by regulating multiple signaling pathways and participates in the regulation of inflammatory signals in macrophages and dendritic cells, thereby affecting the secretion of inflammatory factors. In mouse studies, SIRT1 can reduce inflammatory responses, an effect closely associated with decreased NF-κB activity (7). Based on its anti-inflammatory mechanisms, restoring SIRT1 activity holds promise as a new strategy for the multi-pathway synergistic treatment of CKD. Therefore, this review aims to elucidate the regulatory mechanisms of SIRT1 expression and activity in CKD and its protective role in the core pathological links of CKD (Figure 1), summarize the latest research progress on SIRT1 in treating CKD in recent years, explore the therapeutic potential of SIRT1 in preventing and treating CKD-related complications, and the bright prospects for developing SIRT1 agonists for the treatment of CKD.

**Figure 1:**
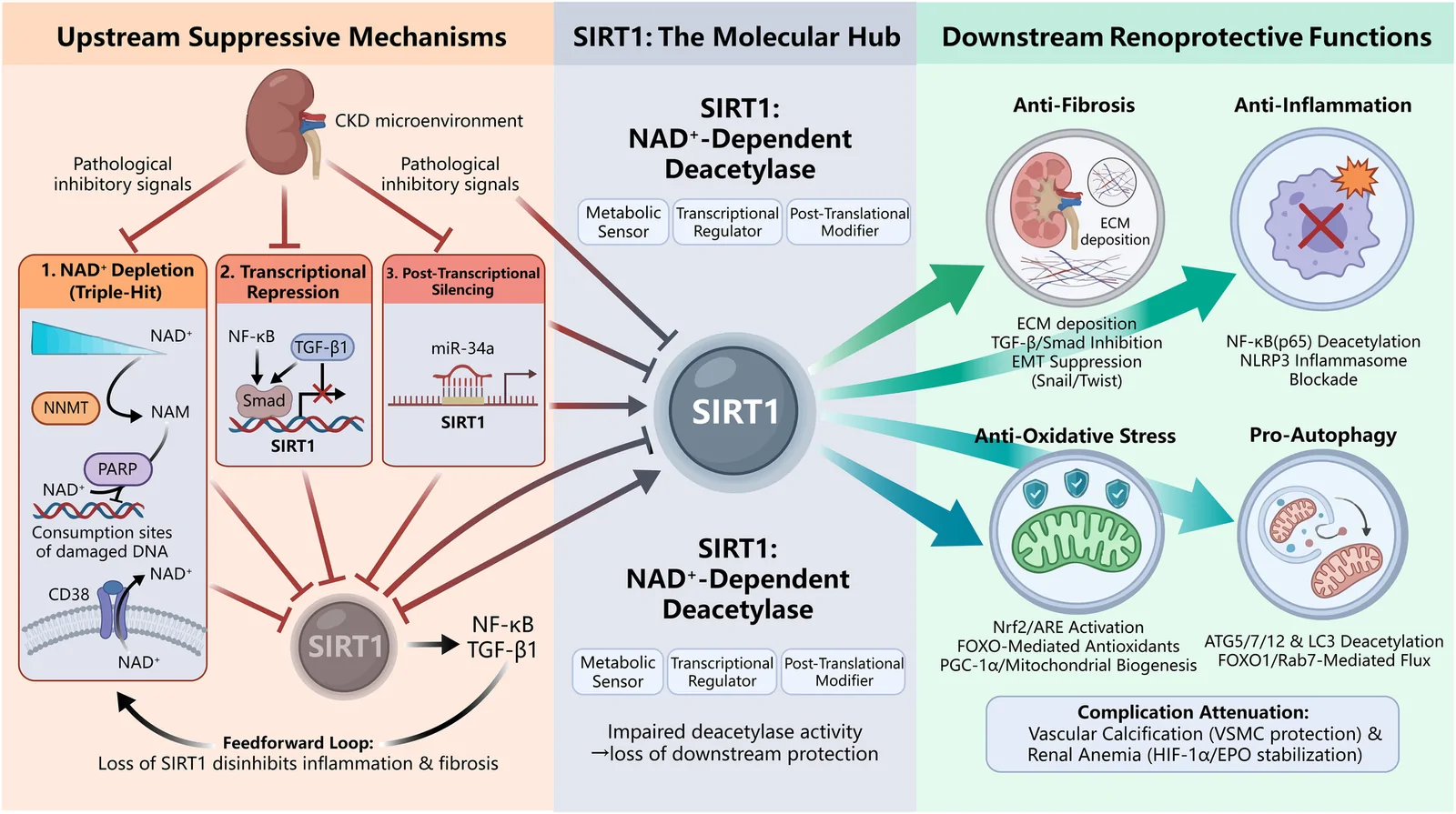
The central molecular Hub of SIRT1 in chronic kidney disease: upstream suppressive mechanisms and downstream renoprotective functions. In the CKD microenvironment, the activity and expression of SIRT1 are strongly suppressed by three pathological signals: first, NAD⁺ depletion (left panel), which is the primary driver of impaired SIRT1 function, involving NAD⁺ diversion mediated by upregulation of NNMT, rapid NAD⁺ consumption induced by hyperactivation of poly (ADP-ribose) polymerase (PARP), and hydrolysis of NAD⁺ and precursors by the transmembrane protein CD38 (8–10); second, transcriptional repression, whereby pro-inflammatory NF-κB and pro-fibrotic signals (TGF-β1/Smad) translocate to the nucleus and directly repress SIRT1 expression at the transcriptional level (11–18); and finally, post-transcriptional silencing, primarily triggered by miR-34a targeting the 3'UTR region of SIRT1 mRNA (19–21). The aforementioned inhibition leads to SIRT1 inactivation, thereby relieving its negative feedback on NF-κB and TGF-β1 and forming a vicious feed-forward loop that exacerbates pathological progression (20, 22–25). As a key NAD⁺-dependent deacetylase, SIRT1 exerts core functions in intracellular metabolic sensing, transcriptional regulation, and post-translational modification (center panel). When SIRT1 is activated (right panel), it exerts significant downstream protective effects through multiple parallel pathways: reducing extracellular matrix (ECM) deposition to counteract fibrosis by deacetylating the TGF-β/Smad complex and inhibiting core EMT factors (Snail/Twist) (11, 26, 27); curbing the inflammatory cascade by deacetylating NF-κB (p65) and blocking NLRP3 inflammasome assembly (28–31); antagonizing oxidative stress by activating the Nrf2/ARE pathway, regulating FOXO-mediated antioxidant enzymes, and enhancing mitochondrial biogenesis via PGC-1α (32–41); and restoring autophagic flux to clear damaged organelles by deacetylating ATG5/7/12 and LC3 and mediating the FOXO1/Rab7 pathway (42–45). Furthermore, SIRT1 antagonizes vascular calcification by protecting vascular smooth muscle cells (VSMCs) and alleviates complications such as kidney anemia by stabilizing HIF-1α to promote erythropoietin (EPO) synthesis (46, 47).

## 2 Expression and regulation of SIRT1 in CKD

### 2.1 Depletion of the core cofactor NAD⁺

The Sirtuin family consists of seven NAD⁺-dependent deacetylases (SIRT1–SIRT7) that exhibit distinct subcellular localization and substrate specificity (32). Among them, SIRT1 (nuclear localization), SIRT3 (mitochondrial localization), and SIRT6 (nuclear localization) have emerged as the most extensively studied members in chronic kidney disease research. These three Sirtuins maintain cellular homeostasis through coordinated actions: SIRT1 primarily regulates gene expression via deacetylation of histones and transcription factors; SIRT3 modulates mitochondrial metabolism and antioxidant defense by deacetylating mitochondrial enzymes; and SIRT6 regulates chromatin structure and DNA repair. Importantly, studies have demonstrated that SIRT1, SIRT3, and SIRT6 synergistically inhibit the TGF-β/Smad and Wnt/β-catenin signaling pathways, both of which are key drivers of kidney fibrosis (11). SIRT1, SIRT3, and SIRT6 also preserve the structural and functional integrity of podocytes (48). This functional network suggests that the net renoprotective effects of SIRT1 activation may be enhanced or complemented by the simultaneous activation of SIRT3 and SIRT6. Conversely, inhibition of SIRT1 may be partially compensated by the activity of SIRT3 or SIRT6, which may explain why SIRT1 knockout models often exhibit milder phenotypes than expected (49). Understanding this Sirtuin network is crucial for developing combination therapeutic strategies that target multiple Sirtuin members to achieve optimal kidney protection.

As an NAD⁺-dependent deacetylase, the activity of SIRT1 is strictly regulated by intracellular NAD⁺ levels. NAD⁺ is an essential cofactor for various regulatory proteins and can ameliorate inflammatory responses, metabolic dysregulation, and extend lifespan (50). The multifaceted functions of NAD⁺ give it a unique position in energy metabolism and various cellular metabolic processes in metabolically active organs such as the heart and kidneys (51). In the state of CKD, the bioavailability of NAD⁺ is significantly decreased, which is a key upstream event leading to the inhibition of SIRT1 activity. The mechanism of NAD⁺ depletion involves a dual imbalance of increased consumption and reduced synthesis. N-methyl-2-pyridone-5-carboxamide (N-Me-2PY) and N-methyl-4-pyridone-3-carboxamide (N-Me-4PY) are final metabolites of NAD⁺ metabolism, reported to be uremic toxins that cause kidney cell injury, and their serum concentrations are higher in CKD patients than in healthy subjects (52). These findings indicate that NAD⁺ metabolism plays a crucial role in the progression of CKD, and the consumption of NAD⁺ is increased under CKD conditions.

Nicotinamide N-methyltransferase (NNMT) is the core enzyme linking NAD⁺ metabolism to the pathological mechanisms of CKD. NNMT catalyzes the transfer of a methyl group from S-adenosylmethionine (SAM) to nicotinamide (NAM), generating S-adenosylhomocysteine (SAH) and methylnicotinamide (MNA); the latter is further metabolized into N-Me-2PY and N-Me-4PY. NNMT participates in the development of age-related non-communicable diseases such as cancer and obesity by consuming methyl donors, reducing NAD⁺ content, and increasing active NAD⁺ metabolites (52, 53). In the kidney, NNMT is highly expressed and regulates the levels of N-Me-2PY and N-Me-4PY (52, 54). As one of the final products of NNMT, N-Me-2PY accumulates in CKD and inhibits poly (ADP-ribose) polymerase (PARP)—a key nuclear enzyme involved in DNA repair regulation (55, 56); its inhibition may delay the recovery of damaged cells and lead to various pathological changes in CKD patients (57, 58). In a mouse model of kidney dysfunction and kidney tissue fibrosis induced by unilateral ureteral obstruction (UUO), NNMT mRNA in mouse kidneys was significantly upregulated, kidney NNMT expression was elevated, accompanied by a decrease in upstream metabolites and an increase in downstream metabolites of NNMT, while SIRT1 expression was significantly reduced. Meanwhile, transgenic mice overexpressing NNMT exhibited more severe tubulointerstitial fibrosis after UUO induction (52). Liver studies also indicate that NNMT promotes inflammation and fibrosis by regulating NAD⁺ and methionine pathways (54). This evidence collectively reveals that under CKD conditions, upregulated NNMT expression leads to excessive consumption of NAD⁺ and simultaneously generates the uremic toxin N-Me-2PY with PARP inhibitory effects. This further interferes with NAD⁺-dependent DNA repair processes, hinders the maturation of nascent DNA strands during DNA replication, and thereby exacerbates genomic instability, cellular senescence, and tubular atrophy (59). NAD⁺ depletion directly inhibits SIRT1 deacetylase activity, weakening its protective functions against oxidation, inflammation, and fibrosis, thereby promoting the progression of CKD.

During the course of CKD, persistent oxidative stress and inflammatory responses induce the massive production of reactive oxygen species (ROS) and reactive nitrogen species (RNS), which can cause oxidative DNA damage in kidney cells (especially kidney tubular epithelial cells) (60). DNA damage activates PARPs, involving them in the DNA repair process; however, this process consumes a vast amount of NAD⁺, which further affects intracellular NAD⁺ levels (51, 61). Studies have found that in cell experiments treated with gamma radiation and neocarzinostatin to induce DNA damage, PARP activity is continuously activated, causing intracellular NAD⁺ levels to plummet to 10%–20% of normal levels within 5–15 min (8). This rapid depletion forces cells to continuously replenish NAD⁺ through *de novo* synthesis or salvage pathways to maintain survival. Even more seriously, NAD⁺ depletion reduces the rate of ATP generation, and ATP is an essential substrate for NAD⁺ resynthesis (62). Under strong DNA damage-inducing conditions, this vicious cycle can form a feedforward loop leading to progressive NAD⁺ depletion, exacerbating the energy metabolism crisis. Kidney tissue in CKD patients is in a state of long-term oxidative stress and low-grade inflammation, and persistent DNA damage maintains PARP activity at a relatively high level, thereby causing continuous NAD⁺ depletion. On one hand, this directly inhibits the deacetylase activity of SIRT1, weakening its kidney protective function; on the other hand, NAD⁺ depletion also exacerbates kidney injury by affecting mitochondrial function, energy metabolism, and cell survival. Furthermore, NAD⁺ biosynthetic pathways may also be impaired in the CKD state. Studies show that nicotinamide phosphoribosyltransferase (NAMPT), the key rate-limiting enzyme of the NAD⁺ salvage pathway, is downregulated in CKD animal models and patients, further exacerbating the deficiency of NAD⁺ (63). The dual mechanisms of insufficient synthesis and increased consumption place the CKD kidney in a state of severe NAD⁺ deficiency and inhibited SIRT1 activity, thereby accelerating disease progression.

In addition to PARPs, CD38, a transmembrane glycoprotein highly expressed in immune cells, is another key enzyme leading to NAD⁺ depletion in CKD (64). Recent studies have revealed that CD38, as the major intracellular NAD⁺ hydrolase, plays a core role in regulating NAD⁺ homeostasis (65). CD38 utilizes NAD⁺ as a substrate to catalyze its hydrolysis into NAM and ADP-ribose (ADPR) (9). Furthermore, CD38 can degrade NAD⁺ precursors—nicotinamide mononucleotide (NMN) and nicotinamide riboside (NR)—extracellularly before they are transported into cells for NAD⁺ biosynthesis (10), thereby further limiting the availability of NAD⁺. Consequently, CD38-mediated NAD⁺ consumption has a direct impact on SIRT1 activity. An important animal experiment demonstrated that CD38 deficiency can attenuate inflammatory responses and apoptosis through the NAD⁺/SIRT1 pathway (66). In that study, CD38 knockout mice exhibited significantly elevated tissue NAD⁺ levels accompanied by enhanced SIRT1 activity, which subsequently alleviated inflammatory responses and apoptosis in various injury models. This finding directly confirms the regulator*y* axis in which CD38 inhibits SIRT1 activity by consuming NAD⁺.

In summary, PARP and CD38 directly inhibit SIRT1 activity by consuming large amounts of NAD⁺, thereby exacerbating oxidative stress, inflammation, and apoptosis, and accelerating CKD progression. Therefore, targeting the inhibition of NNMT, PARP, and CD38, or supplementing NAD⁺ precursors to restore SIRT1 activity, is an important strategy for CKD treatment.

### 2.2 Transcriptional regulation

The expression of SIRT1 is tightly regulated at the transcriptional level, in which nuclear factor-κB (NF-κB) and transforming growth factor-β1 (TGF-β1), as key pro-inflammatory and pro-fibrotic signaling molecules, influence SIRT1 gene transcriptional activity through direct and indirect mechanisms (12). NF-κB is a family of transcription factors composed of p65/RelA, RelB, c-Rel, NF-κB1/p50, and NF-κB2/p52 (13–15), and the SIRT1 gene promoter region contains multiple potential NF-κB binding sites (14), providing a structural basis for direct regulation. However, the regulation of SIRT1 by NF-κB exhibits a complex duality: on one hand, under acute inflammatory stimulation, NF-κB can directly bind to the SIRT1 promoter to promote its transcription. Studies have shown that TNF-α treatment of vascular smooth muscle cells for 8 h can induce SIRT1 upregulation; RelA/p65 overexpression increases SIRT1 mRNA and protein levels at 36 h, while RelA/p65 knockdown attenuates the inductive effect of TNF-α (16). This delayed induction suggests that it may serve as a negative feedback mechanism to limit excessive inflammatory responses. On the other hand, under chronic inflammatory conditions, the inhibition of SIRT1 by NF-κB is not achieved through direct promoter binding, but rather through multiple indirect mechanisms. Yamakuchi et al. (19) identified a potential miR-34a binding site in the 3′UTR of SIRT1, extending from 1,414 bp to 1,439 bp, and confirmed that miR-34a can directly target the SIRT1 3′UTR and inhibit its expression; Li et al. (20) found that the NF-κB complex can directly bind to the miR-34a promoter region and activate miR-34a transcription in a p53-dependent manner. Furthermore, the pro-fibrotic signal TGF-β1 is also involved in the transcriptional repression of SIRT1 (11, 17)—in kidney tubular epithelial cells, TGF-β1 treatment significantly reduces SIRT1 mRNA and protein levels, an effect that can be partially blocked by a Smad3 inhibitor (17, 18), suggesting Smad signaling mediates this process. TGF-β1 may also indirectly inhibit SIRT1 expression by inducing miRNAs such as miR-21 and miR-192, thereby amplifying pro-fibrotic effects (21, 67). During the course of CKD, kidney tissue exists in a long-term pro-inflammatory and pro-fibrotic microenvironment, with sustained activation of NF-κB and TGF-β1. Although compensatory upregulation of SIRT1 may occur in the early stages of inflammation, with disease progression, the indirect inhibitory effect mediated by the NF-κB-miR-34a axis becomes dominant, leading to a sustained decline in SIRT1 expression. This weakens its anti-inflammatory and antioxidant functions, resulting in disinhibition of NF-κB and TGF-β1 signaling and exacerbating inflammation and fibrosis (20, 22–25). Notably, factors such as aging, hyperglycemia, and oxidative stress can further inhibit SIRT1 expression (49, 68–70), which may be an important reason why the elderly and diabetic patients are more prone to CKD progression. Therefore, the transcriptional regulation of SIRT1 is complex: NF-κB can directly activate SIRT1 transcription in the short term, but also indirectly inhibit SIRT1 expression in the long term by inducing miRNAs such as miR-34a. In the chronic inflammatory setting of CKD, NF-κB sustains SIRT1 downregulation via indirect mechanisms (miR-34a upregulation, ROS accumulation, and NAD⁺ depletion), rather than direct transcriptional repression. This SIRT1 deficiency removes negative feedback on NF-κB, forming a vicious feed-forward loop that perpetuates kidney inflammation and fibrosis (71). Consequently, inhibiting inflammatory signaling pathways and the secretion of pro-inflammatory factors is an important measure for kidney protection during CKD progression (Figure 2).

**Figure 2:**
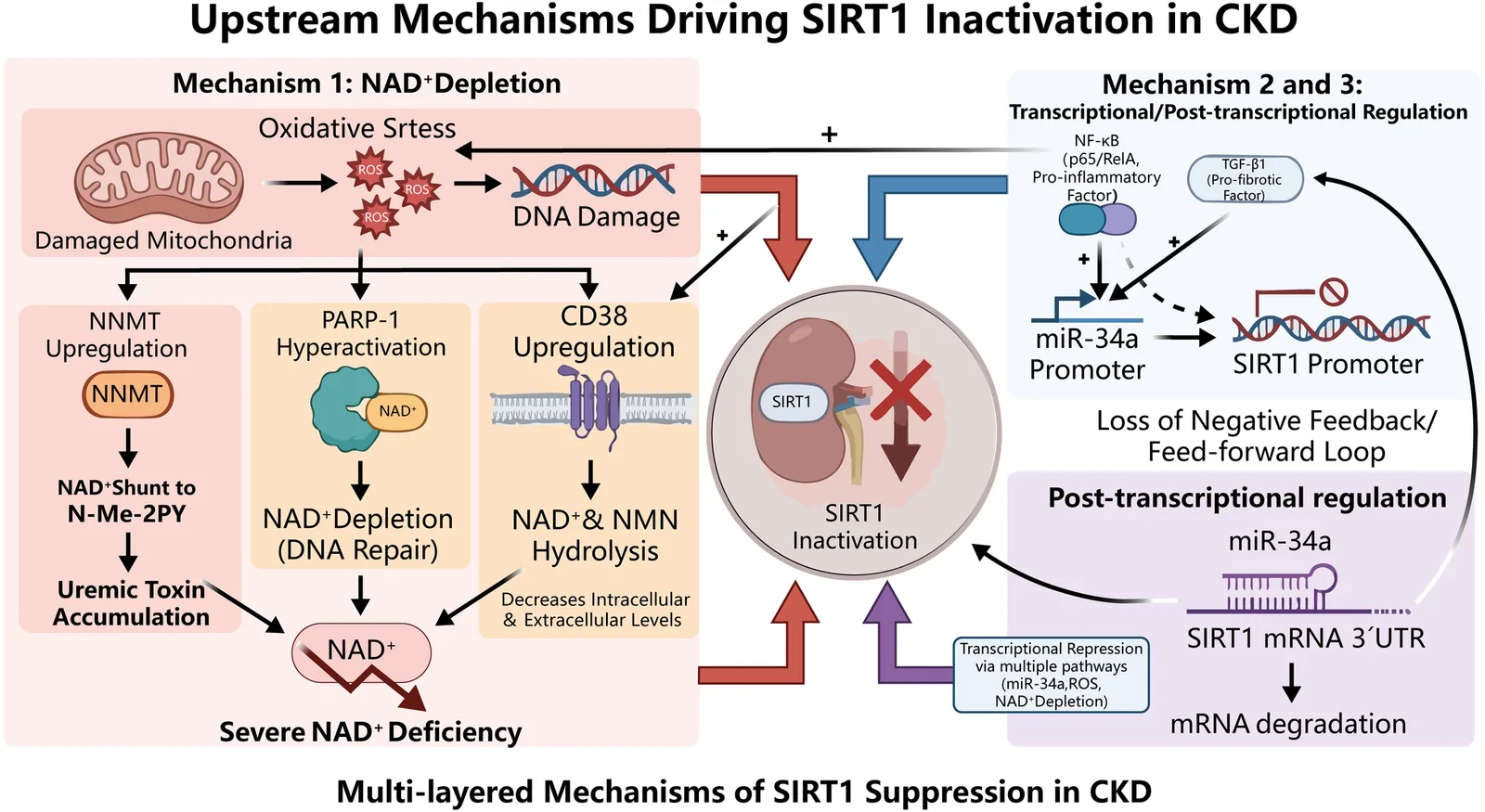
Multi-layered mechanisms of SIRT1 suppression in CKD. The inhibition of SIRT1 activity in CKD is primarily attributed to two core mechanisms: NAD⁺ depletion (left) and regulation at transcriptional/post-transcriptional levels (right). In Mechanism 1, oxidative stress-induced DNA damage hyperactivates poly (ADP-ribose) polymerase-1 (PARP-1), which drastically consumes NAD⁺ during participation in DNA repair (8, 51, 60, 61); meanwhile, upregulated nicotinamide N-methyltransferase (NNMT) expression diverts NAD⁺ metabolism and generates the uremic toxin N-Me-2PY that inhibits PARP repair function (52, 54–56); furthermore, enhanced hydrolase activity of the transmembrane glycoprotein CD38 further degrades intracellular and extracellular NAD⁺ and its precursor NMN; these three hits collectively lead to severe NAD⁺ deficiency (9, 10, 64–66). In Mechanisms 2 and 3, the pro-inflammatory factor NF-κB (p65/RelA) and the pro-fibrotic factor TGF-β1 translocate to the nucleus and indirectly suppress SIRT1 expression via miR-34a upregulation, ROS accumulation, and NAD⁺ depletion at the transcriptional level (12–18, 72). Additionally, NF-κB can bind to the miR-34a promoter to activate its expression; the resulting miR-34a subsequently targets the 3'UTR region of SIRT1 mRNA, inducing degradation of the target mRNA and forming long-term post-transcriptional inhibition (19, 20, 23–25, 73). The aforementioned transcriptional inhibition and inactivation of SIRT1 further relieve the negative feedback inhibition on NF-κB and TGF-β1, creating a vicious feedforward loop that exacerbates the pathological progression of CKD (20, 23–25, 71, 73).

## 3 Protective role of SIRT1 in the core pathological mechanisms of CKD

Cell type-specific factors to consider in SIRT1-mediated kidney protection. It must be recognized that SIRT1 exerts distinct functions in different kidney cell types, a factor with important implications for the interpretation of its net renoprotective effects. In kidney tubular epithelial cells, SIRT1 activation suppresses apoptosis, promotes autophagy, and inhibits EMT through deacetylation of FOXO3a, Smad3, and Snail (49). In podocytes, SIRT1 maintains the integrity of the glomerular filtration barrier by deacetylating FOXO4 and inhibiting apoptosis; podocyte-specific SIRT1 deletion exacerbates glomerulosclerosis and proteinuria in aged mice (70). In macrophages, SIRT1 promotes anti-inflammatory M2 polarization and inhibits NLRP3 inflammasome activation (74). In VSMCs, SIRT1 counteracts vascular calcification by inhibiting osteogenic transdifferentiation and cellular senescence (46). However, studies have reported that in kidney interstitial fibroblasts, SIRT1 activation promotes fibroblast activation and exacerbates fibrosis via EGFR and PDGFR signaling pathways, revealing a paradoxical pro-fibrotic effect in this cell type (75). This cellular heterogeneity underscores the critical importance of developing cell-specific targeting strategies for SIRT1-based therapies in CKD.

### 3.1 Antagonizing kidney fibrosis

Kidney fibrosis is the terminal pathological process of CKD, characterized by excessive deposition of extracellular matrix (ECM) leading to scarring (75, 76). The development of kidney fibrosis involves multiple types of kidney cells, and its mechanisms involve various factors, including tubular epithelial cell injury, inflammatory cell infiltration, and myofibroblast activation (77). During the progression of kidney fibrosis, multiple cellular processes occur simultaneously and are interrelated. These processes include the activation of mesangial cells and fibroblasts, epithelial-mesenchymal transition (EMT) of kidney tubular epithelial cells, infiltration of monocytes/macrophages and T cells, as well as apoptosis (78). TGF-β, Wnt, Notch, and Hedgehog signaling pathways are major regulatory pathways for kidney fibrosis, influencing its occurrence and development independently or through interactions, with primary influencing factors including cellular hypoxia, inflammation, aging, drugs, metabolic disorders, and hypertension (79). These factors indicate an impaired healing response of kidney tissue following chronic injury.

SIRT1 plays a key antagonistic role in the process of kidney fibrosis by targeting the TGF-β1/Smad signaling pathway. The TGF-β1/Smad signaling pathway is a core pathway mediating kidney fibrosis, liver fibrosis, and pulmonary fibrosis (80, 81). As a key pro-fibrotic mediator, TGF-β1 drives progressive kidney fibrosis by stimulating excessive ECM production and inhibiting its degradation (80); its signaling process is: TGF-β1 binds to TGF-βRII, prompting phosphorylation of TGF-βRI, which in turn phosphorylates Smad2/3; the phosphorylated Smad2/3 forms a heterotrimeric complex with Smad4 and translocates to the nucleus to initiate the transcription of pro-fibrotic genes (such as collagen I, collagen IV, and fibronectin) (80). The pro-fibrotic activity of TGF-β depends on the acetylation modification of Smad3 and Smad4, with acetylation enhancing their induced transcriptional activity (26, 27, 72). SIRT1 directly inhibits TGF-β signaling by deacetylating Smad3 and Smad4. In UUO and 5/6 nephrectomy models, upregulation of SIRT1 by resveratrol or SRT3025 significantly reduces kidney inflammatory responses and decreases the accumulation of collagen IV and fibronectin through Smad3 deacetylation (26, 27, 72). Furthermore, overexpression of SIRT1 in kidney tubular cells can prevent the progression of acute kidney injury to kidney interstitial fibrosis through Smad4 deacetylation and reduce the accumulation of matrix metalloproteinase-7 (MMP7) (72), which is associated with kidney aging and fibrosis (82, 83).

SIRT1 antagonizes the progression of kidney fibrosis by inhibiting EMT through deacetylating EMT-related transcription factors (72, 75). EMT is a process in which kidney tubular epithelial cells lose their epithelial phenotype and acquire mesenchymal cell characteristics, and is regarded as one of the key cellular mechanisms of kidney fibrosis. Snail and Twist are key transcription factors of EMT; they drive the transformation of epithelial cells into mesenchymal cells by inhibiting the expression of epithelial markers such as E-cadherin and inducing the expression of mesenchymal markers such as α-smooth muscle actin (α-SMA) (84, 85). SIRT1 maintains epithelial phenotype stability by deacetylating Snail and Twist, thereby promoting their degradation or inhibiting their transcriptional activity. A study by Song et al. (86) revealed the molecular mechanism by which fatty acid oxidation (FAO) regulates EMT via the SIRT1/STAT3/Twist1 pathway: CPT1A-mediated FAO activation can upregulate SIRT1 expression; SIRT1 subsequently deacetylates STAT3 and inhibits its pro-fibrotic activity, ultimately downregulating Twist1 expression and inhibiting the EMT process in kidney tubular epithelial cells. Notably, FAO inhibition precedes the occurrence of EMT, suggesting that FAO deficiency is an upstream event driving EMT. FAO is a key regulator of cellular senescence; the ablation of FAO can trigger p53-mediated senescence in human fibroblasts, while enhanced FAO activity can alleviate replicative senescence (87). In the context of kidney fibrosis, cellular senescence induced by FAO inhibition further promotes fibrosis progression, whereas SIRT1 overexpression or autophagy activation can alleviate the senescent phenotype caused by FAO inhibition (87).

It is noteworthy that the role of SIRT1 in kidney fibrosis exhibits significant cell-type dependence, a characteristic that is crucial for evaluating its potential as an anti-fibrotic target (75). In kidney tubular epithelial cells, SIRT1 exerts a clear protective effect by deacetylating Smad3/Smad4 to inhibit TGF-β signaling and suppress EMT (26). In podocytes, SIRT1 exerts an anti-apoptotic effect through deacetylation of FOXO4, thereby maintaining the integrity of the glomerular filtration barrier (88). However, in kidney interstitial fibroblasts, the role of SIRT1 appears to be opposite. Ponnusamy (89) et al. found that in a UUO-induced kidney fibrosis model, the SIRT1 activator SRT1720 promoted kidney fibroblast activation and aggravated fibrosis, with the underlying mechanism involving activation of EGFR and PDGFR signaling pathways. This finding suggests that SIRT1 activation in fibroblasts may exert pro-fibrotic effects, in stark contrast to its protective role in kidney tubular epithelial cells. Therefore, when evaluating the potential of SIRT1 as an anti-fibrotic target, its differential roles across various kidney cell types must be comprehensively considered, and priority should be given to developing targeting strategies with cell-type selectivity.

In summary, SIRT1 inhibits the TGF-β1/Smad signaling pathway by deacetylating Smad3 and Smad4, integrates FAO metabolic status, STAT3 signaling, and Twist1 transcriptional regulation, reduces ECM deposition, inhibits EMT, antagonizes kidney fibrosis progression, and improves the status of CKD (Figure 3).

**Figure 3:**
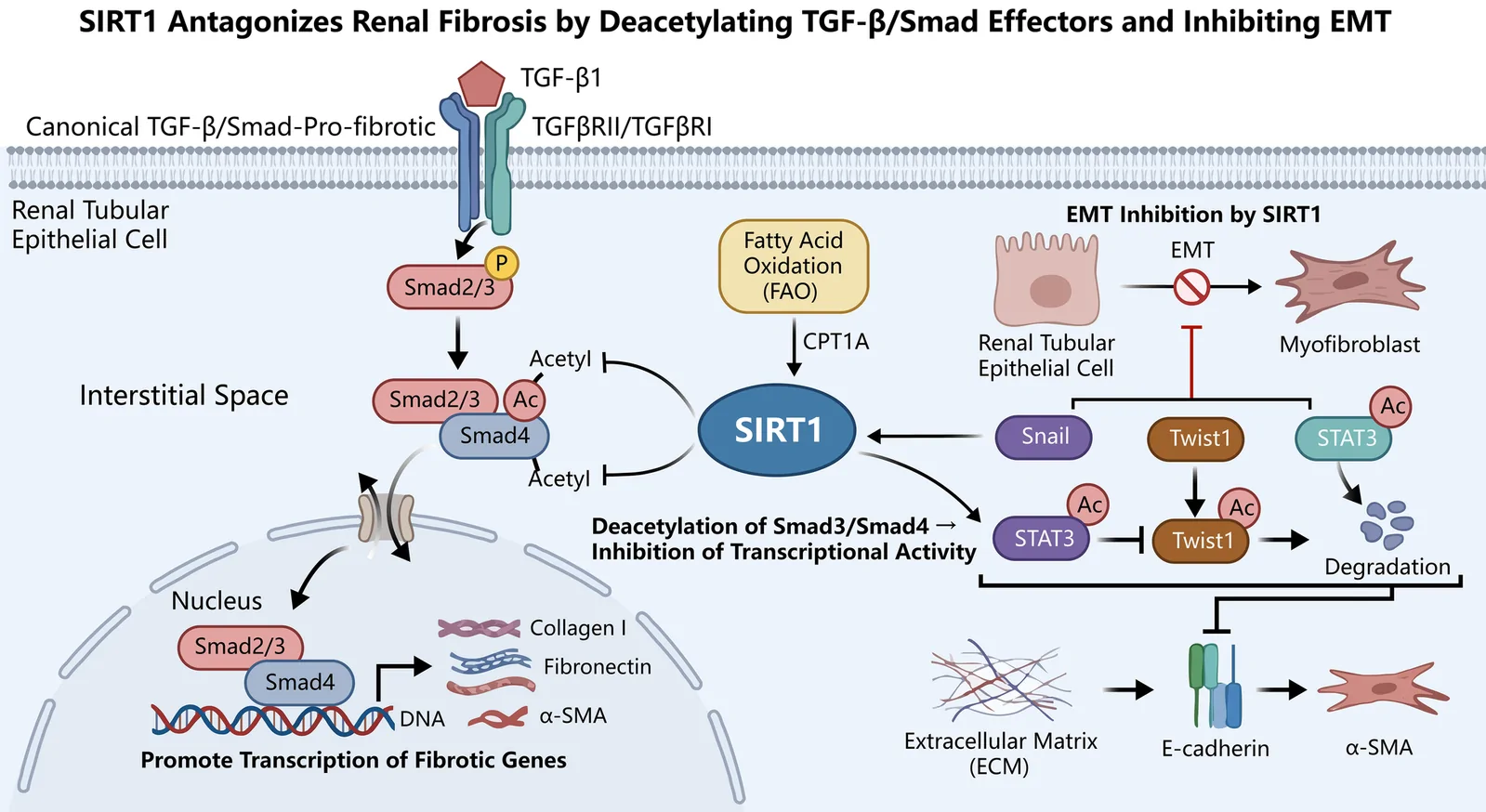
SIRT1-mediated protection against kidney fibrosis and EMT. At the cellular and interstitial levels, SIRT1 exerts anti-kidney fibrosis effects through dual inhibitory pathways. In the classic pro-fibrotic pathway (left), the TGF-β1 ligand binds to cell membrane receptors (TGFβRII/TGFβRI) and induces Smad2/3 phosphorylation; phosphorylated Smad2/3 subsequently binds to Smad4 to form a complex and translocates to the nucleus, thereby initiating the transcription of pro-fibrotic genes such as collagen I, fibronectin, and α-smooth muscle actin (α-SMA) (80, 81, 84, 85). SIRT1 significantly blocks the transcriptional activity of this complex by directly deacetylating the acetyl groups (Ac) of Smad3 and Smad4, thereby reducing the excessive deposition of extracellular matrix (ECM) in the interstitial space (26, 27, 72, 75, 76). In the pathway inhibiting epithelial-mesenchymal transition (EMT) (right), activated by CPT1A-mediated fatty acid oxidation (FAO), SIRT1 deacetylates the downstream transcription factor STAT3, which in turn downregulates the expression or promotes the degradation of core EMT regulators such as Twist1 and Snail (84–86). This mechanism effectively maintains the expression of epithelial markers (e.g., E-cadherin) in kidney tubular epithelial cells and prevents the aberrant expression of interstitial markers such as α-SMA as well as transformation into myofibroblasts.

### 3.2 Alleviating inflammatory responses

SIRT1 plays a key alleviating role in kidney inflammation in CKD by targeting the NF-κB signaling pathway and the NLRP3 inflammasome (90). NF-κB is one of the most extensively studied pathways and is core to mediating inflammatory responses; in a resting state, it exists in the cytoplasm as a complex bound to members of the IκB family. Upon inflammatory activation, IκB kinase (IKK) mediates the phosphorylation and degradation of IκB, and the released NF-κB (primarily composed of p65 and p50) translocates to the nucleus to initiate the transcription of pro-inflammatory cytokine genes (91, 92). SIRT1 inhibits the NF-κB pathway through multiple mechanisms: first, SIRT1 directly deacetylates the lysine 310 residue of the p65 subunit, inhibiting its transcriptional activity, thereby reducing the expression of inflammatory factors such as IL-1β, IL-6, and TNF-α (28, 29); second, SIRT1 can inhibit p65 nuclear translocation and reduce its DNA-binding capacity (93); furthermore, SIRT1 can also inhibit IκB degradation, maintaining NF-κB in an inactive state in the cytoplasm (94). In a CKD model induced by repeated low-dose cisplatin, SIRT1 downregulation leads to elevated p65 acetylation levels and sustained NF-κB activation, whereas SIRT1 activation significantly reduces p65 acetylation and alleviates inflammation and fibrosis responses (24). In addition to NF-κB, SIRT1 also negatively regulates the NLRP3 inflammasome—a polymeric protein complex that mediates pyroptosis by sensing danger signals to activate caspase-1 and promoting the maturation and release of IL-1β and IL-18 (95). NLRP3 can be activated by ROS, exacerbating the inflammatory cascade and mitochondrial dysfunction and forming a vicious cycle (96). SIRT1 directly inhibits its activation by deacetylating NLRP3, while simultaneously promoting mitochondrial biogenesis and reducing mitochondrial ROS production by activating PGC-1α, and indirectly eliminating upstream signals for NLRP3 activation by upregulating the expression of antioxidant enzymes such as HO-1, SOD, and CAT via activation of the Nrf2 pathway (30, 31). In diabetic nephropathy models, hyperglycemia induces macrophage infiltration and inflammatory factor release, inhibits the Nrf2 pathway, and exacerbates oxidative stress, whereas SIRT1 activation can inhibit the NLRP3 inflammasome and improve kidney function (30, 31, 97). In macrophages, SIRT1 promotes the polarization shift from pro-inflammatory M1 to anti-inflammatory M2 phenotype and inhibits NLRP3 inflammasome activation (74). In kidney tubular epithelial cells, SIRT1 suppresses NF-κB signaling by deacetylating the K310 site of p65, thereby reducing the production of IL-1β, IL-6, and TNF-α (3). These cell type-specific anti-inflammatory effects of SIRT1 collectively contribute to the resolution of kidney inflammation in CKD. Thus, SIRT1 curtails the kidney inflammatory cascade at multiple levels by dually blocking NF-κB-mediated transcriptional activation and NLRP3-mediated inflammasome assembly, making it a highly promising intervention target for anti-inflammatory therapy in CKD.

### 3.3 Reducing oxidative stress

SIRT1 plays a multi-level protective role in oxidative stress injury in CKD by activating the Nrf2/ARE antioxidant pathway, regulating the FOXO transcription factor family, and enhancing PGC-1α-mediated mitochondrial biogenesis (32, 33, 98, 99). Under physiological conditions, Nrf2 binds to the cytoplasmic inhibitor Keap1 and remains in an inactive state; upon oxidative stimulation, Nrf2 dissociates from Keap1 and translocates to the nucleus, initiating the transcription of downstream antioxidant enzyme genes (such as HO-1, SOD-1) (100). SIRT1 enhances the stability and transcriptional activity of Nrf2 through deacetylation, thereby activating the Nrf2/ARE pathway. Studies have shown that resveratrol significantly eliminates excessive ROS production induced by advanced glycation end products (AGEs) and downregulates the expression of fibronectin (FN) and TGF-β1 by activating the SIRT1-Nrf2-ARE pathway; whereas SIRT1 depletion blocks the activation of the Nrf2/ARE pathway and reverses the protective effects of resveratrol (34, 101). In models of diabetic nephropathy, resveratrol treatment increased the levels of SIRT1 and Nrf2 in kidney tissues, improving antioxidant capacity and kidney dysfunction. Furthermore, a positive feedback loop is formed between SIRT1 and the Nrf2-ARE pathway, and the protective effect provided by t-BHQ activating the Nrf2-ARE pathway in db/db mice is partially dependent on SIRT1 (102).

In addition to the Nrf2 pathway, SIRT1 enhances cellular antioxidant defense capabilities by regulating the deacetylation of the FOXO transcription factor family (32). FOXO (including FOXO1, FOXO3a, FOXO4) are key transcription factors that regulate the expression of antioxidant enzyme genes (such as MnSOD, catalase CAT). Under oxidative stress conditions, the binding of SIRT1 to FOXO3 and FOXO4 increases, regulating FOXO transcriptional activity through deacetylation (35, 103). Similar to the regulation of p53 by SIRT1, after SIRT1 deacetylates FOXO, it shifts the cellular response triggered by FOXO from inducing apoptosis to inducing cell cycle arrest and resisting oxidative stress, upregulating the expression of the DNA repair gene GADD45 and the manganese superoxide dismutase gene MnSOD (35–37), thereby providing protection against oxidative damage caused by ROS. As an energy sensor, AMPK is activated during cellular energy depletion or metabolic stress, subsequently promoting SIRT1 activity; SIRT1 enhances FOXO transcriptional activity through deacetylation and upregulates the expression of antioxidant enzymes such as SOD and CAT, forming an AMPK-SIRT1-FOXO antioxidant axis (104, 105). SIRT1 enhances cellular resistance to oxidative stress by regulating pathways involving AMPK, FOXO1, and FOXO3a, which mitigate ROS production and lipid peroxidation (38). For example, resveratrol ameliorates kidney tubular oxidative stress injury in diabetic rat models by regulating the SIRT1/FOXO3a pathway (106); aminophylline inhibits the progression of chronic kidney failure in 5/6 nephrectomized rat models by regulating SIRT1/AMPK/mTOR-mediated autophagy (107).

SIRT1 also enhances mitochondrial function and reduces mitochondrial ROS production by regulating PGC-1α. PGC-1α is a master transcription factor regulating mitochondrial biogenesis and antioxidant defense. By co-activating nuclear respiratory factors (NRF1 and NRF2), it upregulates the expression of mitochondrial transcription factor A (TFAM). TFAM translocates to the mitochondrial matrix to stimulate mitochondrial DNA replication and mitochondrial gene expression, promoting mitochondrial biogenesis (38–40). Mitochondrial biogenesis can be defined as the growth and division of existing mitochondria, maintaining mitochondrial homeostasis and energy metabolism by regulating the generation of new mitochondria, thereby protecting kidney cells from oxidative damage (108). SIRT1 enhances PGC-1α transcriptional activity by increasing PGC-1α expression and decreasing its acetylation level (41). NAD⁺, as a substrate of SIRT1, affects PGC-1α function by regulating SIRT1 expression (109). Experiments using a mouse ischemia-reperfusion model and HK-2 cells indicate that PGC-1α overexpression inhibits inflammation and oxidative stress, thereby protecting the kidney from injury (110). The biological functions of PPARs rely on the synergistic activation by PGC-1α. PPARs regulate the expression of genes involved in energy metabolism and inflammation, further expanding the metabolic regulatory network of the SIRT1-PGC-1α axis (111, 112). The decline in mitochondrial function is closely related to aging-related pathological states such as type 2 diabetes and Alzheimer's disease (113), whereas activation of the SIRT1-PGC-1α axis can improve mitochondrial dynamics and energy metabolism, maintaining mitochondrial homeostasis (114, 115).

In summary, SIRT1 constructs a multi-level antioxidant defense network at both the transcriptional and mitochondrial levels through the synergistic effects of three pathways: activating the Nrf2/ARE pathway, regulating the FOXO transcription factor family, and enhancing PGC-1α-mediated mitochondrial biogenesis. This network alleviates oxidative stress damage in the kidneys of CKD and delays disease progression (Figure 4).

**Figure 4:**
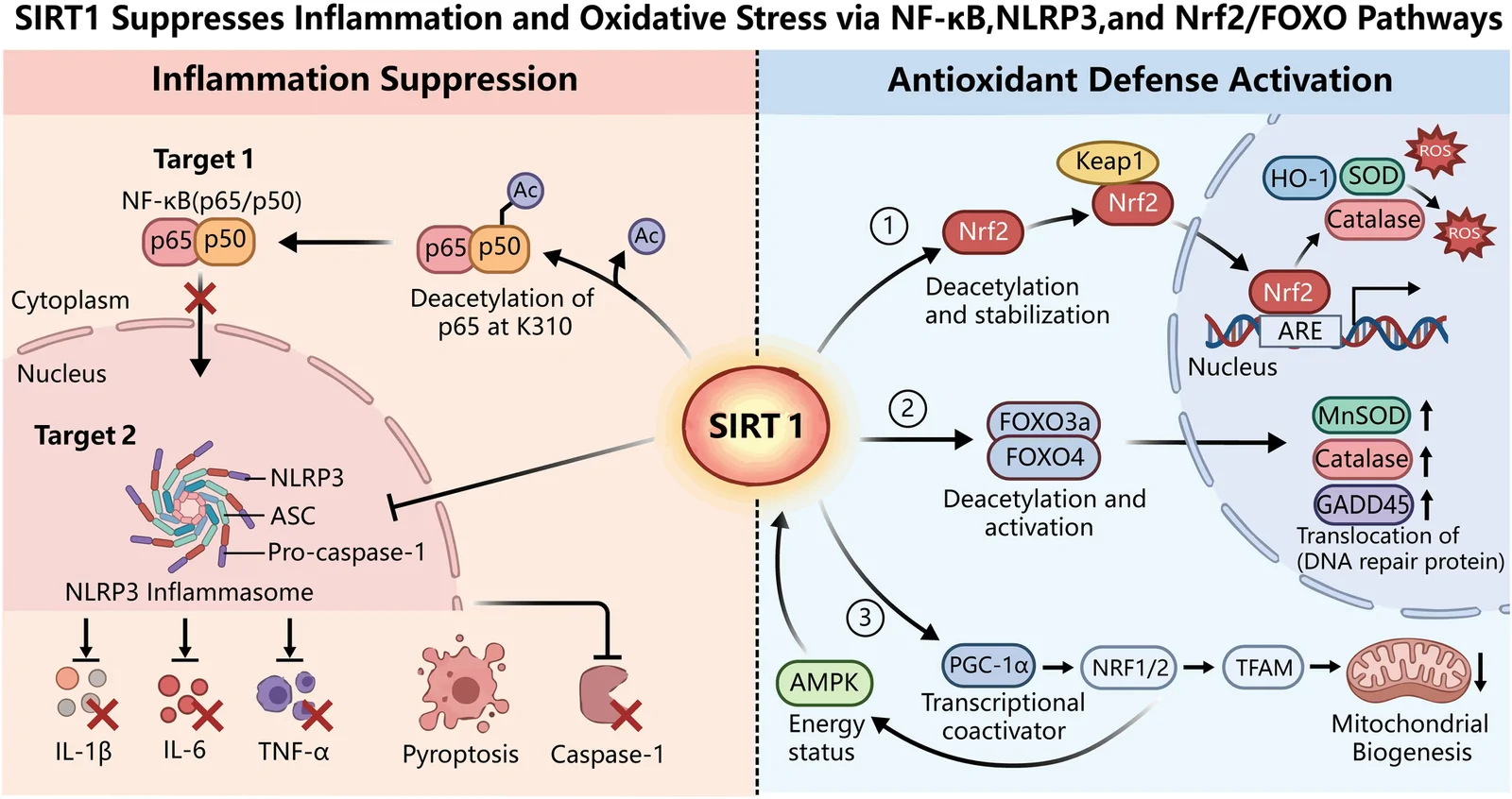
Multi-target anti-inflammatory and antioxidant mechanisms of SIRT1. As a central hub of intracellular metabolism and stress response, SIRT1 coordinates dual defense mechanisms: anti-inflammatory (left panel) and antioxidant (right panel). Regarding inflammation suppression, SIRT1 inhibits the expression of downstream pro-inflammatory cytokines (IL-1β, IL-6, TNF-α) by deacetylating the p65 subunit of the NF-κB complex in the cytoplasm (specifically targeting the K310 site) and blocking its nuclear translocation (28, 29, 91–93); meanwhile, SIRT1 directly represses the assembly of the NLRP3 inflammasome composed of NLRP3, ASC, and Pro-caspase-1, thereby blocking caspase-1 activation and the subsequent pyroptosis process (30, 31, 95, 96). In terms of antioxidant defense, SIRT1 functions through three parallel pathways (32, 98, 99, 101): ① deacetylating and stabilizing Nrf2, promoting its dissociation from the inhibitor protein Keap1 and translocation into the nucleus to bind antioxidant response elements (ARE), thereby upregulating heme oxygenase-1 (HO-1), superoxide dismutase (SOD), and catalase to scavenge reactive oxygen species (ROS) (34, 100, 101); ② deacetylating and activating the FOXO3a and FOXO4 transcription factors to upregulate the expression of MnSOD, Catalase, and the DNA repair protein GADD45 (35–37); ③ deacetylating the transcriptional coactivator PGC-1α to drive mitochondrial biogenesis via the downstream NRF1/2-TFAM axis, reducing ROS generation originating from damaged mitochondria (38–41). Additionally, activation of AMPK, the cellular energy status sensor, can upregulate SIRT1, forming a positive closed-loop network that maintains redox homeostasis (38, 104, 105).

### 3.4 Promoting autophagy

Autophagy defect is an important feature of CKD, and SIRT1, as a key autophagy regulatory molecule, plays a core role in maintaining kidney cell homeostasis by directly deacetylating core autophagy components and indirectly regulating autophagy-related transcription factors (42, 116, 117). The regulation of autophagy by SIRT1 is mainly achieved through two pathways: First, SIRT1 directly deacetylates autophagy-related proteins (ATGs) to promote autophagosome formation. Under nutrient-rich conditions, E1A binding protein p300 (EP300) inhibits the formation of the ATG5-ATG12-ATG16 complex by acetylating ATG5, ATG7, and ATG12, thereby preventing autophagosome elongation; whereas under starvation conditions, SIRT1 directly deacetylates ATG5, ATG7, and ATG12 in an NAD⁺-dependent manner, activating the complex and promoting autophagic vesicle elongation (42, 43). Additionally, SIRT1 also regulates microtubule-associated protein light chain 3 (LC3)—a key protein in autophagosome formation. Under starvation conditions, SIRT1 rapidly deacetylates the K49 and K51 sites of nuclear LC3-I; deacetylated LC3-I interacts with the *δ*-opioid receptor (DOR) and is exported together to the cytoplasm, where it binds to ATG7 and conjugates with phosphatidylethanolamine (PE) to form LC3-II, embedding into the isolation membrane to participate in autophagosome formation (44). Second, SIRT1 upregulates the expression of autophagy and lysosome-related genes by deacetylating the transcription factors FOXO1 and TFEB. FOXO1 is a key transcription factor regulating autophagy; under nutrient-deficient conditions, SIRT1 expression increases in the kidneys of cadmium-exposed rats, promoting FOXO1 nuclear translocation by deacetylating it, thereby upregulating Rab7 expression—Rab7 is an important protein mediating the late fusion of autophagosomes with lysosomes; it is recruited on the autophagosome membrane and plays an important regulatory role in autophagosome maturation, intracellular cargo transport, and the docking and fusion of autophagosomes with lysosomes (45). This study activated SIRT1 using the SIRT1 agonist SRT2104, deacetylated FOXO1, restored Rab7 expression, and alleviated autophagosome-lysosome fusion, thereby relieving cadmium-induced autophagy inhibition and alleviating kidney injury in experimental rats. Experiments have also demonstrated that resveratrol promotes Rab7 expression, reduces p62 accumulation, enhances autophagic flux, and improves myocardial oxidative damage in diabetic mice by activating the SIRT1/FOXO1 pathway (118). In vascular endothelial cells, resveratrol similarly promotes autophagy through the SIRT1/FOXO1 pathway (119). Functional experiments further confirmed the autophagy-regulating role of SIRT1: in a sepsis-associated acute kidney injury (SAKI) model, after the SIRT1 agonist SRT1720 activated SIRT1, mitochondrial swelling was alleviated, the number of autophagosomes increased, and Beclin1 and LC3-II protein expression was upregulated; conversely, the SIRT1 inhibitor Ex527 exacerbated mitochondrial swelling and reduced autophagosome formation (120). These results indicate that SIRT1 protects kidney cells by promoting autophagic flux, and its mechanism involves the direct deacetylation of core autophagy components and the upregulation of autophagy-related gene expression mediated by the transcription factor FOXO1. In the context of CKD, SIRT1 downregulation and activity inhibition lead to autophagy defects, obstructed clearance of damaged organelles and protein aggregates, and accelerated kidney fibrosis and loss of kidney function.

## 4 SIRT1 and CKD-related complications

### 4.1 Chronic kidney disease-mineral and bone disorder (CKD-MBD) and vascular calcification

Vascular calcification is a core complication of CKD-MBD and an independent risk factor for cardiovascular events and all-cause mortality in patients with CKD (121, 122). SIRT1 antagonizes CKD-MBD-related vascular calcification by inhibiting the osteogenic differentiation of vascular smooth muscle cells (VSMCs), delaying cellular senescence, and alleviating endoplasmic reticulum stress and oxidative stress through multiple signaling pathways (46). Smooth muscle cells possess phenotypic plasticity; under normal physiological conditions, they exist in a contractile phenotype, expressing contractile-related proteins such as smooth muscle α-actin (SMαA), SM-22α, and smooth muscle myosin heavy chain (SM-1/SM-2) to maintain vascular tone and extracellular matrix homeostasis (123). However, in the context of CKD, injurious factors such as hyperphosphatemia, inflammation, and oxidative stress induce the transformation of VSMCs from a contractile to an osteogenic phenotype via the Pit-1 cotransporter. This transformation is characterized by the loss of contractile protein markers and the acquisition of osteogenic proteins such as Runt-related transcription factor 2 (Runx2), osteopontin, osteocalcin, alkaline phosphatase (ALP), and type I collagen, as well as the release of calcifying vesicles (124, 125). SIRT1 exerts an anti-calcification effect by inhibiting Runx2 activity through deacetylation modification and reversing osteogenic phenotypic transdifferentiation (46). Studies have confirmed that SIRT1 activators can alleviate VSMC mineralization by upregulating the expression of calcification inhibitors osteoprotegerin (OPG) and osteopontin (OPN) (126).

SIRT1 is able to bind to p300 and inhibit β-catenin nuclear translocation, thereby preventing osteogenic activity and vascular calcification. The histone acetyltransferase p300 serves as a coactivator for various transcription factors, and p300-mediated acetylation of β-catenin can promote β-catenin signaling activation (127, 128). However, under hyperglycemic conditions, SIRT1 expression is downregulated, rendering it unable to inhibit p300-mediated β-catenin acetylation and GSK-3β-mediated β-catenin phosphorylation. This leads to the binding of β-catenin to TCF/LEF, driving the transcription of Runx2 and bone morphogenetic proteins (BMPs), and promoting osteogenic differentiation (129). Low levels of SIRT1 are barely able to prevent this process, whereas the SIRT1 activator resveratrol can inhibit hyperphosphatemia-induced VSMC osteogenic transdifferentiation, apoptosis, and senescence (130).

Cellular senescence is another important driver of vascular calcification. In senescent endothelial cells, endothelial nitric oxide synthase (eNOS) expression is downregulated, making cells susceptible to oxidative stress damage. Caloric restriction (CR) delays endothelial cell senescence by inducing eNOS expression accompanied by increased SIRT1 levels (131). Nitric oxide (NO) is synthesized in endothelial cells via the conversion of L-arginine and is catalytically generated by eNOS through calcium-calmodulin control (132). Endothelium-derived NO possesses vasodilatory and antioxidant properties, helping to protect blood vessels from atherosclerosis and oxidative stress (133). FOXO transcription factors are important factors in maintaining endothelial morphology and exert antioxidant protective effects by inducing endothelial cell apoptosis and inhibiting angiogenesis (134–136). SIRT1 overexpression prevents oxidative stress injury in endothelial progenitor cells (EPCs) by inhibiting FoxO3a-mediated apoptosis (137), and EPCs play an important role in repairing vascular injury. In VSMCs, SIRT1 activates their antioxidant properties by upregulating FOXOs, thereby delaying cellular senescence. microRNA-34a promotes VSMC senescence and mineralization by downregulating the AXL receptor tyrosine kinase and SIRT1, further confirming the central role of SIRT1 in anti-senescence and anti-calcification (130). In addition, pro-inflammatory adipokines released by dysfunctional perivascular adipose tissue (PVAT) mediate medial calcification and arterial stiffness (138). SIRT1 improves PVAT adipokine release and increases adiponectin secretion, the latter interacting with FOXO1 to combat oxidative stress and inflammatory arterial injury (138). Endoplasmic reticulum stress and oxidative stress are also involved in the process of vascular calcification (139), and SIRT1 alleviates these stress responses through deacetylation, protecting VSMC homeostasis.

In summary, multiple factors such as osteogenic differentiation of VSMCs, cellular senescence, endoplasmic reticulum stress, and oxidative stress induce vascular calcification, leading to increased vascular wall stiffness, hypertension, left ventricular hypertrophy, insufficient myocardial tissue perfusion, and elevated cardiovascular mortality. This is a non-negligible risk factor for the progression of CKD to ESKD. However, SIRT1 attenuates the process of vascular calcification through multiple targets and pathways by affecting endothelial NO bioavailability, senescence of endothelial cells and VSMCs, osteogenic phenotypic transdifferentiation, VSMC apoptosis, extracellular matrix deposition, and PVAT inflammatory response.

### 4.2 Kidney anemia

In patients with CKD, insufficient EPO synthesis and disordered iron metabolism are traditional causes of kidney anemia (140, 141). HIF-1 is a transcription factor that mediates cellular adaptation to hypoxia levels by regulating the expression of genes involved in angiogenesis, metabolism, and cell survival (142). The regulation of HIF-1 activity by SIRT1 primarily depends on its deacetylation function; this modification acts directly on the HIF-1α subunit, thereby affecting its protein stability and transcriptional activation capacity (47). The interaction between SIRT1 and HIF-1α is particularly important under hypoxic conditions, as the stabilization and activation of HIF-1α are crucial for cellular adaptation (47, 143, 144). Joo et al. demonstrated that the accumulation of HIF-1α was not observed in SIRT1-deficient clonal cells under hypoxic conditions (47). However, under normoxic conditions, HIF-1α accumulation could still be observed even in the absence of SIRT1, indicating that SIRT1 is crucial for HIF-1α stability in a hypoxic environment. Notably, the addition of a SIRT1 inhibitor to the culture medium led to the rapid degradation of HIF-1α. Furthermore, studies have found that SIRT1 directly interacts with the inhibitory domain of HIF-1α, protecting it from degradation (47). SIRT1 is a key molecule for maintaining the hypoxic adaptive response; its functional deficiency not only disrupts the stability of the hypoxic state but also directly impairs the synthesis and release of erythropoietin (EPO) mediated by HIF signaling.

Notably, SIRT1 overexpression induced by SGLT2 inhibitor treatment can inhibit ferroptosis and prevent the harmful consequences caused by excessive cytoplasmic iron (145). Recent studies indicate that SIRT1 controls ferroptosis by influencing multiple target genes, including p53, whose deacetylation can inhibit ferroptosis (146). In fact, substantial evidence suggests that SIRT1 plays a crucial role in the regulation of ferroptosis. More specifically, SIRT1 inhibits ferroptosis by deacetylating p53, thereby alleviating damage caused by calcium oxalate (CaOx) crystals (147, 148). Another target of SIRT1 is nuclear factor erythroid 2-related factor 2 (NRF2), which serves as another key factor in inhibiting ferroptosis by preventing lipid peroxidation. SIRT1 can exert anti-ferroptotic effects by activating NRF2; NRF2 is a key regulator of the antioxidant stress response and also influences iron metabolism by enhancing ferritin formation and inducing hepcidin (146).

In summary, the chronic hypoxic microenvironment of CKD leads to a reduction in HIF-1α, affecting its capabilities such as protecting blood vessels and maintaining cell survival, impairing the synthesis and release of EPO, and accelerating the progression of kidney anemia, while simultaneously exacerbating CKD. Conversely, SIRT1 activates HIF-1α by deacetylating it under hypoxic conditions, blocking this process, and simultaneously inhibiting cellular ferroptosis through various means such as deacetylating p53, thereby alleviating kidney anemia.

## 5 Therapeutic potential and strategies targeting SIRT1

Given the critical protective role of SIRT1 in regulating core pathological processes of CKD (such as fibrosis, inflammation, oxidative stress, and autophagy), targeted activation of SIRT1 has emerged as a highly promising therapeutic strategy for delaying CKD progression. Currently, research on SIRT1 agonists primarily focuses on synthetic natural compounds, small-molecule agonists, and NAD⁺ precursor supplementation.

Natural polyphenolic compounds are the earliest discovered and most extensively studied SIRT1 agonists. Resveratrol, a natural polyphenol found in plants such as grapes and Polygonum cuspidatum, is the most deeply investigated natural SIRT1 agonist (149); however, its clinical application faces significant challenges. Studies explicitly indicate that the clinical application of resveratrol is limited by its “low oral bioavailability.” This issue stems from its rapid phase II metabolism (glucuronidation and sulfation), resulting in extremely low concentrations of the parent drug in the systemic circulation (150, 151). Furthermore, resveratrol exhibits low potency and insufficient specificity toward SIRT1, making it difficult to achieve ideal target regulatory effects (152, 153). To address this bottleneck, researchers are developing resveratrol derivatives to improve pharmacokinetic properties. Ciccone et al. reported that although the resveratrol derivative 3d, obtained through computational virtual screening, showed weaker SIRT1 activation effects than resveratrol at the *in vitro* isolated enzyme level, the effects of both were comparable in human umbilical vein endothelial cells, suggesting that this derivative may possess stronger transmembrane ability and intracellular target accessibility (154). Natural products such as quercetin and Salvia miltiorrhiza injection have demonstrated kidney protective effects by activating SIRT1 in animal models (155, 156). Quercetin increases SIRT1 nuclear activity in a rat model of cadmium chloride-induced kidney injury, inhibiting endoplasmic reticulum stress through deacetylation of Xbp-1s and eIF2α, and its protective effect can be completely abolished by the SIRT1 inhibitor EX-527 (155). In a kidney fibrosis model, quercetin attenuates senescence of kidney tubular epithelial cells by activating the SIRT1/PINK1/mitophag*y* axis (156). The active component of Salvia miltiorrhiza, salvianolic acid B, can upregulate SIRT1 expression and activate autophagy, improving kidney function in rats with kidney fibrosis (157); salvianolate induces autophagy in podocytes of rats with membranous nephropathy by activating the AMPK/SIRT1/PGC-1α pathway (158), but most of these studies are in the preclinical stage and lack systematic human pharmacokinetic and safety data.

SRT2104 is a first-generation non-resveratrol synthetic SIRT1 activator with superior specificity and pharmacokinetic properties compared to resveratrol (159, 160). Multiple Phase I and Phase II clinical trials have been completed, providing important data for evaluating its application in humans. The first-in-human trial in healthy volunteers showed that SRT2104 exhibited dose-dependent but sub-proportional increases in plasma concentration after oral administration. The single-dose range was 0.03 g to 3.0 g, and the accumulation factor was less than 3-fold after 7 days of repeated dosing. The mean bioavailability was approximately 14%, and the mean clearance was approximately 400 mL/min. The time to peak concentration (Tmax) was 2–4 h, and the elimination half-life was 15–20 h (161, 162). Notably, food significantly affects drug exposure; when taken with a high-calorie meal, exposure can increase up to 4-fold, suggesting that standardized administration conditions are necessary in clinical application (161). A 28-day dosing study in elderly volunteers (60–80 years) further confirmed these pharmacokinetic characteristics; after once-daily dosing of 0.5 g and 2.0 g, the increase in exposure was less than dose-proportional, with high inter-individual variability (162). In multiple clinical trials, SRT2104 demonstrated good safety and tolerability. In the first-in-human trial, no serious adverse events were observed in any dose group (161). In a 28-day Phase II trial in patients with type 2 diabetes, there was no significant difference in the incidence of adverse events between the treatment groups and the placebo group (163). SRT1720 is another widely studied synthetic SIRT1 activator, currently explored primarily at the preclinical stage. In the C57BL/6J mouse model, SRT1720 (100 mg/kg/day) extended average lifespan by 8.8% and reduced mortality by 27.0% (164). There are currently no published clinical trial data on SRT1720 in humans.

Increasing intracellular NAD⁺ levels by supplementing NAD⁺ precursors (NMN, NR) to indirectly activate SIRT1 is another important strategy (165). Compared to direct SIRT1 activators, NAD⁺ precursors function by replenishing substrates rather than directly activating the enzyme, theoretically offering broader metabolic regulatory effects (73). Currently, NMN and NR have shown protective effects in various animal models of aging-related diseases (166, 167), and some small-scale human clinical trials have been conducted (167). However, their pharmacokinetic characteristics, safety, and efficacy in patients with CKD lack systematic research and require further validation (Table 1).

**Table 1:** Comparison of SIRT1-targeted therapeutic agents.

Category	Natural polyphenols	Synthetic SIRT1 agonists	NAD⁺ precursors	Novel SIRT1 modulators
Representative Agents	Resveratrol, Quercetin	SRT2104, SRT1720	NMN, NR	SRT2379 (discontinued)
Mechanism of Action	Direct SIRT1 activation (controversial); indirect via AMPK/Nrf2	Allosteric SIRT1 activation	NAD⁺ supplementation → indirect SIRT1 activation	SIRT1 activation
Bioavailability/ PK	Extremely low oral bioavailability (<5 ng/mL plasma); rapid Phase II metabolism	SRT2104: F∼14%, Tmax 2–4 h, t½ 15–20 h; subproportional exposure	Orally bioavailable; rapidly converted to NAD⁺	Not reported
Selectivity	Low (multiple off-target effects)	High (SRT2104 highly selective)	Broad (activates multiple NAD⁺-dependent enzymes)	Moderate
Clinical Development Stage in CKD	Preclinical only	SRT2104: Phase I-II completed; SRT1720: Preclinical	Preclinical; small-scale human trials in other diseases	Discontinued (Phase I)
Major Translational Barriers	Extremely low bioavailability; poor target specificity; controversial mechanism; no CKD patient data	High PK variability; subproportional exposure; disconnect between preclinical and clinical efficacy; did not meet primary glycemic endpoint; no CKD efficacy data	No CKD-specific clinical data; unknown safety profile in CKD patients	Failed to reduce inflammatory cytokines in human endotoxemia model
Refs	(149–154)	(89, 152, 159–164)	(73, 165–167)	-

CKD, chronic kidney disease; ECM, extracellular matrix; EMT, epithelial–mesenchymal transition; F, bioavailability; NMN, nicotinamide mononucleotide; NR, nicotinamide riboside; PK, pharmacokinetic; t₁/₂, elimination half-life; Tmax, time to maximum concentration; UUO, unilateral ureteral obstruction. Reference numbers correspond to the citation list in the main text.

In summary, therapeutic strategies targeting SIRT1 present distinct pathways in clinical translation: natural activator resveratrol is limited by the bottleneck of low oral bioavailability, and the optimization of its derivatives is still under exploration; the synthetic activator SRT2104 has completed multiple Phase I-II clinical trials, demonstrating good safety and certain metabolic improvement effects, but its pharmacokinetic characteristics (sub-proportional exposure, high inter-individual variability) may affect the stability of efficacy, and its primary endpoint for blood glucose control was not met; development of SRT2379 was terminated due to lack of efficacy in inflammation models, suggesting that the anti-inflammatory effects of SIRT1 activators in humans may require higher target occupancy or optimized molecular design; the NAD⁺ precursor strategy has theoretical advantages but lacks clinical evidence in the CKD population.

## 6 Challenges and difficulties

### 6.1 The clinical translation of targeting SIRT1 still faces multiple challenges and difficulties

First, tissue specificity and cell type heterogeneity are core challenges. SIRT1 may play different roles in different kidney cell types (podocytes, kidney tubular epithelial cells, endothelial cells, immune cells), and systemic activation of SIRT1 may produce unintended effects in certain tissues (89, 168). In podocytes, SIRT1 exerts an anti-apoptotic effect by deacetylating FOXO4; in proximal tubular cells, SIRT1 promotes autophagy and reduces apoptosis by deacetylating FOXO3a (168). However, SIRT1 activators can promote kidney fibroblast activation and exacerbate kidney fibrosis, suggesting that the role of SIRT1 is cell type-dependent (89). Therefore, developing kidney cell type-selective SIRT1 targeting strategies is crucial to avoid unintended effects caused by systemic activation.

Second, the “double-edged sword effect” of SIRT1 needs to be carefully evaluated. As research progresses, controversial conclusions have emerged regarding the role of SIRT1 in different disease stages and different cell types (75). For example, in kidney fibrosis research, SIRT1 may play distinct roles in different kidney cell types: it exerts protective effects in kidney tubular epithelial cells and podocytes, but in kidney interstitial fibroblasts, SIRT1 activation can promote fibrosis through the EGFR and PDGFR pathways. Systemic activation of SIRT1 may produce complex or even counteracting outcomes due to its differential effects across cell types (89). Excessive activation of SIRT1 may promote tumorigenesis or interfere with normal repair processes in certain situations (169, 170). Therefore, a more detailed resolution of the spatiotemporal dynamics of SIRT1 in different stages of CKD (early inflammatory phase vs. late fibrotic phase) is needed.

Third, the pharmacological limitations of existing agonists urgently need to be overcome. Natural agonists (such as resveratrol) suffer from issues such as low bioavailability, rapid metabolism, and broad targets (152, 171); synthetic agonists (such as SRT1720), although improved in selectivity, still require systematic evaluation of their long-term safety, optimal dosages, and administration regimens (172, 173). From the perspective of chemical structure optimization, developing novel SIRT1 agonists with higher selectivity and kidney-enriching properties is an important direction for the future. Additionally, although NAD⁺ precursor (NMN, NR) supplementation strategies can indirectly activate SIRT1, their efficacy in CKD patients still requires large-scale clinical validation (6). A major bottleneck in translating SIRT1-targeted therapies into clinical practice for CKD lies in the disconnect between preclinical efficacy endpoints and clinically meaningful outcomes. To date, nearly all preclinical studies have relied on surrogate endpoints such as serum creatinine (Scr), blood urea nitrogen (BUN), urinary albumin-to-creatinine ratio (UACR), and histologic quantification of kidney interstitial fibrosis area to assess therapeutic efficacy. Although these biomarkers are valuable for proof-of-concept studies, they do not directly reflect the hard clinical endpoints that ultimately determine patient prognosis, namely progression to ESKD requiring dialysis or transplantation, the incidence of major adverse cardiovascular events (MACE), and all-cause mortality. The lack of long-term data linking SIRT1 activation to these hard endpoints represents a critical gap in the existing evidence base. Furthermore, most preclinical studies have relatively short timeframes (typically 4–12 weeks), which are insufficient to adequately model the decades-long natural course of human CKD. This temporal disconnect raises an important question: whether the renoprotective effects observed in animal models can be sustained over timeframes more relevant to clinical CKD management. To bridge this gap in translational research, future preclinical studies should extend observation periods and evaluate outcome measures that translate more directly to human clinical endpoints. Additionally, clinical trials designing SIRT1-targeting agents should prioritize the inclusion of hard endpoints, while recognizing that improvements in surrogate biomarkers may not necessarily translate into clinically meaningful benefits for patients.

Fourth, patients with CKD of different etiologies and stages may require individualized treatment strategies. Under the backgrounds of different etiologies such as diabetic nephropathy, hypertensive nephropathy, and glomerulonephritis, differences may exist in the expression and regulatory mechanisms of SIRT1; research shows that urinary SIRT1 levels are decreased in hypertensive patients (whether or not complicated by diabetes), and are lowest in patients complicated with diabetes (174). Patients with early-stage CKD may benefit more from SIRT1 activation, while late-stage patients may have a limited response to intervention due to irreversible fibrosis (89). Therefore, future research needs to establish precision stratification strategies based on biomarkers [such as urinary miR-200a-3p (175) and serum SIRT1 levels (176)].

Fifth, combination therapeutic strategies are worthy of in-depth exploration. The combined application of SIRT1 agonists with existing therapeutic agents (such as RAAS inhibitors and SGLT2 inhibitors) may produce synergistic effects (177); for example, SGLT2 inhibitors have been confirmed to enhance autophagy and alleviate inflammation by activating SIRT1 (178, 179), and the combination of both may achieve multi-pathway synergistic intervention.

Sixth, the spatiotemporal duality of SIRT1 during CKD progression warrants particular attention. In the early inflammatory phase of CKD, SIRT1 activation may serve as a compensatory protective mechanism, thereby limiting excessive inflammation and oxidative stress (75). However, in the advanced fibrotic stage, the net benefit of SIRT1 activation may be undermined by its paradoxical pro-fibrotic effects on fibroblasts and irreversible tissue damage (89). This temporal duality suggests that the optimal therapeutic window for SIRT1 activation may lie in the early-to-mid stages of CKD, whereas late-stage intervention may yield limited efficacy. Future research should employ temporally controlled genetic and pharmacological models to investigate the efficacy of SIRT1-targeted therapy across different disease stages.

### 6.2 Clinical significance of SIRT1 in CKD and prospects for nephrology practice

Although SIRT1 remains primarily a research target rather than an established clinical therapeutic modality in the field of CKD, the evidence summarized in this review has multiple implications for current nephrology clinical practice and future clinical research. First, the close association between SIRT1 dysfunction and key pathological processes of CKD (fibrosis, inflammation, oxidative stress, and autophagic dysfunction) raises the hypothesis that monitoring SIRT1 activity or its downstream effectors could, with further validation, serve as an adjunct for assessing disease activity and progression (176) However, this remains a research concept and requires validation through large prospective cohort studies before any clinical application can be considered. Although serum SIRT1 levels have shown potential as biomarkers in certain studies, their clinical utility requires validation through large prospective cohort studies before incorporation into routine clinical application. Second, the identification of SIRT1 as a central hub integrating multiple protective pathways highlights the potential benefits of naturally enhancing SIRT1 activity through lifestyle interventions. Preclinical studies have demonstrated that caloric restriction and regular physical exercise can upregulate SIRT1 expression, suggesting a hypothesis that these lifestyle interventions could exert kidney-protective effects as adjunctive strategies in CKD management (32); however, these measures must be carefully tailored to each patient's nutritional status and functional capacity when implemented. Third, the cell type-specific and stage-dependent duality of SIRT1 function has important implications for patient selection in future clinical trials. The optimal candidates for SIRT1-targeted therapy are likely to be patients in the early-to-moderate stages of CKD who have not yet developed irreversible fibrosis, as well as those in whom inflammation or oxidative stress predominates as the underlying pathological mechanism. For patients with advanced fibrosis, or those in whom fibroblast activation is the predominant pathogenic mechanism, systemic SIRT1 activation may confer only limited benefit or even pose potential harm. Fourth, from a practical perspective, the most immediately feasible approach to harnessing the SIRT1 pathway in CKD may be through NAD⁺ supplementation rather than direct SIRT1 activation. NAD⁺ precursors such as NMN and NR have been demonstrated to be safe in short-term human studies in non-CKD populations, and are commercially available as dietary supplements. However, their efficacy and safety specifically in the CKD population have not been established, and their use should be considered strictly within the context of clinical investigation rather than as an established clinical strategy (51). The advantage of this approach lies in its ability to simultaneously support multiple NAD⁺-dependent pathways, including SIRT1, PARP, and CD38, thereby conferring broader metabolic benefits. Finally, the translational barriers identified in this review, particularly the reliance on surrogate endpoints in preclinical studies and the lack of definitive clinical outcome data, should inform the design of future clinical trials. Regulatory agencies and trial sponsors should consider prioritizing the inclusion of clinically meaningful endpoints, such as ESKD incidence, cardiovascular events, and mortality, rather than relying solely on biomarker changes, in order to definitively establish the therapeutic value of SIRT1-targeted strategies in CKD.

## 7 Future directions and solutions

Research in this field should be advanced synergistically across three dimensions: mechanistic elucidation, drug development, and clinical translation. At the mechanistic level, it is necessary to utilize conditional gene knockout mouse models to precisely dissect the spatiotemporal-specific functions of SIRT1 in different kidney cell types (such as kidney tubular epithelial cells, podocytes, and immune cells) and across different stages of CKD progression (the early inflammatory phase and the late fibrotic phase) (180–182), and to deeply explore the synergistic regulatory networks between SIRT1 and other sirtuin family members such as SIRT3 and SIRT6 (49, 183). Regarding drug development, efforts should be focused on virtual screening and rational design based on the crystal structure of SIRT1 to develop a new generation of small molecule agonists with higher selectivity and kidney targeting (184), and to explore nanotechnology-based targeted delivery systems (185), with the aim of increasing local drug concentration in the kidney, enhancing therapeutic efficacy, and reducing systemic side effects. In the realm of clinical translation, there is an urgent need to conduct large-scale, multi-center prospective cohort studies to systematically validate the correlation between SIRT1 expression levels and prognostic indicators in CKD patients (such as the rate of eGFR decline, time to onset of ESKD, and risk of cardiovascular events) (186), while also exploring the potential of SIRT1-related miRNAs in urinary exosomes as non-invasive diagnostic biomarkers (175, 187). Meanwhile, future research should also explore the therapeutic potential of dual or pan-Sirtuin activation strategies. Given that SIRT1, SIRT3, and SIRT6 exert complementary functions in maintaining cellular homeostasis, simultaneous activation of multiple Sirtuins may confer superior renoprotective effects compared with SIRT1 activation alone (49). For instance, combination therapy with SIRT1 and SIRT3 activators could concurrently target nuclear gene regulation and mitochondrial metabolism, thereby addressing multiple pathological pathways in CKD progression (188). Furthermore, the development of isoform-selective Sirtuin modulators will facilitate more precise elucidation of the independent and synergistic roles of each Sirtuin member in CKD.

## 8 Conclusion

CKD has become a global public health issue; its progressive kidney fibrosis is the key pathological process leading to ESKD, and existing therapeutic measures are still difficult to effectively reverse this process. This review systematically elaborates on the core protective role of SIRT1 as an NAD⁺-dependent deacetylase in CKD and its multi-layered regulatory network.

The activity of SIRT1 is subject to precise control via multi-dimensional regulation in CKD. At the upstream level, NAD⁺ depletion is the primary driver of impaired SIRT1 function, involving a triple-hit mechanism: NNMT upregulation-mediated nicotinamide shunting and the generation of the uremic toxin N-Me-2PY; drastic NAD⁺ consumption and energy crisis triggered by PARP overactivation; and the degradation of NAD⁺ precursors due to enhanced CD38 hydrolase activity. Meanwhile, pro-inflammatory and pro-fibrotic signals such as NF-κB and TGF-β1 directly inhibit SIRT1 expression at the transcriptional level and form long-term inhibition at the post-transcriptional level via miRNAs such as miR-34a. This constitutes a positive feedback loop wherein inflammatory signals upregulate miRNAs, leading to SIRT1 downregulation and consequently exacerbating inflammation. This multi-layered regulatory imbalance places the CKD kidney in a state of severely suppressed SIRT1 activity.

At the functional level, SIRT1 antagonizes the core pathological links of CKD by integrating multiple protective pathways: antagonizing kidney fibrosis by deacetylating Smad3/Smad4 and inhibiting the EMT process; curbing the inflammatory cascade by dually blocking NF-κB transcriptional activity and NLRP3 inflammasome assembly; constructing a multi-layered antioxidant defense network by activating the Nrf2/ARE pathway, regulating FOXO transcription factors, and enhancing PGC-1α-mediated mitochondrial biogenesis; and restoring autophagic flux and clearing damaged organelles by directly deacetylating core autophagy elements (ATG5/7/12, LC3) and indirectly regulating FOXO1-mediated autophagy-related gene expression. However, the role of SIRT1 is markedly cell-type-dependent: in kidney tubular epithelial cells and podocytes, it delays CKD progression by antagonizing fibrosis, inflammation, oxidative stress, and autophagic defects. Conversely, in kidney interstitial fibroblasts, SIRT1 activation may promote fibrosis through the EGFR and PDGFR pathways. Therefore, the net effect of SIRT1 depends on the cell type in which it is activated and the disease stage, making it crucial to develop cell-type-selective SIRT1-targeting strategies. Regarding complications, SIRT1 antagonizes vascular calcification by inhibiting VSMC osteogenic-like transformation and senescence, and improves kidney anemia by stabilizing HIF-1α to promote EPO synthesis and inhibiting ferroptosis.

In terms of therapeutic strategies, the synthetic SIRT1 agonist SRT2104 has completed multiple phase I-II clinical trials; although it did not meet expectations on primary endpoints such as glycemic control, it demonstrated good safety and improvement effects on biomarkers such as lipid metabolism and mitochondrial function. The natural agonist resveratrol is limited by extremely low bioavailability, and its structural optimization is currently underway. The NAD⁺ precursor strategy possesses theoretical advantages but lacks clinical evidence in the CKD population. Currently, the disconnect between the pharmacokinetic characteristics of SRT2104 observed in human clinical studies (sub-proportional increase in exposure, high inter-individual variability) and its preclinical kidney protective effects, as well as the functional heterogeneity of SIRT1 in different kidney cell types and disease stages, constitute the major obstacles for translation from the laboratory to the clinic.

In summary, SIRT1 integrates multi-level functions including metabolic sensing, transcriptional regulation, and post-translational modification, serving as a key molecular hub linking upstream pathological signals to downstream organ damage in CKD. Targeting SIRT1 activation offers the unique advantage of simultaneously intervening in fibrosis, inflammation, oxidative stress, and defective autophagy. Future research should focus on: utilizing conditional gene knockout models to precisely dissect the functional heterogeneity of SIRT1 in different kidney cell types and at different stages of CKD; developing a new generation of agonists with higher selectivity and kidney targeting based on the crystal structure of SIRT1; and conducting rigorously designed clinical trials to verify the efficacy and safety of SIRT1 agonists in CKD patients. Ultimately, precise regulation of SIRT1 activity holds promise for providing CKD patients with a novel multi-target synergistic therapeutic strategy.
